# An empirical research based on spatial–temporal evolution of high-quality tourism development in Fujian Province of China

**DOI:** 10.1371/journal.pone.0315221

**Published:** 2024-12-13

**Authors:** Ying Ke, Min Yang, Yajun Xie

**Affiliations:** 1 College of Business Administration, Fujian Jiangxia University, Fujian, China; 2 School of Big Data, Fuzhou University of International Studies and Trade, Fujian, China; Centro de Investigaciones Biologicas del Noroeste SC, MEXICO

## Abstract

The high-quality development of tourism is crucial to the sustainable development of regional economy. To evaluate high-quality tourism development, this paper has developed an index system with 6 second-level indicators and 24 third-level indicators and used methods of entropy-weight, AHP, and TOPSIS to empirically assess the high-quality tourism development of 9 cities in Fujian Province. According to the results, there are obvious regional differences in the development of high-quality tourism in Fujian Province. From 2016 to 2019, the overall development trend of cities in Fujian Province was consistent, showing a steady upward trend. Green development in tourism has the best performance, which was less affected by the COVID-19. Fuzhou and Xiamen contribute most to the tourism development of Fujian Province, while other cities are lagging behind for various reasons and the lack of innovation and shared development are two of them. Based on the results of the research, we put forward the following suggestions: Fujian should coordinate the planning of the province’s green eco-tourism resources to maximize the use of resources. It should combine the advantages of the primary, secondary and tertiary industries and fully develop both advanced regions and under-developed regions. It should also explore areas of potential growth in the tourism sector, such as Sanming, Longyan, and Nanpin, by strengthening digital innovation and sharing resources with Xiamen, Fuzhou, Quanzhou and other highly-developed tourism regions.

## Introduction

At present, China’s economic development has entered a new phase, characterized by its economic shift from rapid growth to high-quality development. High-quality development aims at meeting “the people’s ever-growing needs for a better life”. As the first of the “five happiness industries”, tourism boosts the revenue of the economy, creates thousands of jobs, develops the infrastructures of a country, and plants a sense of cultural exchange between foreigners and citizens. It improves people’s quality of life by enhancing people’s well-being, sense of belonging, and connectedness to nation [1]. A high-quality development of the tourism sector should be well-organized, large-scale, fast, efficient, and safe. In March 2022, the National People’s Congress (NPC) and the National Committee of the Chinese People’s Political Consultative Conference (CPPCC) were held. High-quality development of tourism was high on the agenda of the "two sessions." According to the country’s 14th Five-year Plan (2021–2025), the high-quality development of tourism should focus on deep integration of culture and tourism to create a unique experience centered on Chinese culture for travelers. The document said that to achieve the goal, it is important to provide diversified tourism products while protecting the environment through industrial chain and digital innovation, shared development, and tourism regulations. Meanwhile, China’s 14th Five-Year Plan aims to inaugurate a structural change from intensive accumulation to innovation-driven and to enhance the competitiveness of tourism and achieve sustainable development.

Despite massive flows of tourists clearly benefit the local economy, the pursuit of economic benefits by tourism entities also leads to overtourism [2]. Scholars have further investigated the quality development of tourism in the post-Covid-19 period, as the recovery and transformation of the tourism sector have become urgent priorities [3]. As the consensus has been reached on importance of improving the quality of the tourism sector, all regions have step up the quality development of tourism [4]. Fujian Province seized the opportunity of economic recovery to promote the recovery and high-quality development of cultural tourism. It adopted various measures, including increasing the investment in the tourism sector and issuing supportive documents such as *the Opinions of the People’s Government of Fujian Province on Promoting the High-Quality Development of Tourism* and *the Action Plan for Promoting the High-Quality Development of the Culture and Tourism Economy in Fujian Province* (2022–2025). However, only a handful of studies evaluated the role of high-quality development on sustainable tourism [5], and even fewer studies focused on the high-quality tourism development in Fujian Province.

This study first provided the definition of high-quality tourism development, and then introduced the concepts involved in high-quality tourism, such as coordinated and green development, tourism innovation, resources sharing and openness. Moreover, it used the index system to evaluate high-quality tourism development in Fujian Province and adopted entropy-weight method, AHP, and the TOPSIS method to solve the indicators’ weights and approaching degree of progress (CDP). Finally, this study examined the characteristics of high-quality development of tourism in Fujian Province from the most basic dimensions of time and space. Recommendations were given based on the regional differences to promote the high-quality development of tourism in Fujian Province.

The main contribution in this paper is the use of the evaluation index system to objectively show degree of high-quality tourism development. Current evaluation index system adopted by previous literature only focuses on one single factor without considering synergistic effect of the both "quantity" and "quality" developments. The new system in this paper adopted multiple perspectives, such as "supports, means, orientation, motivation, goals, and directions", and it used the percentages and growth rates to reflect development results. Therefore, this paper can reflect the influencing factors of high-quality tourism in a comprehensive way. Moreover, this study has conducted thorough investigation and multi-dimensional analysis of the high-quality tourism development of 9 cities in Fujian Province. It quantitatively measured the characteristics of high-quality tourism in different development phases and compared the regional differences in Fujian Province. By accurately reflecting Fujian tourism’s development patterns and trends. It offers broader, deeper and more differentiated insights for policy-makers to promote high-quality tourism development in Fujian Province and other regions with similar conditions.

## Literature review

Tourism comprises the activities of persons travelling to and staying in places outside their usual environment for not more than one consecutive year for leisure, business, or other purposes [6]. High-quality tourism brings both economic and social benefits [7]. Many scholars have investigated the high-quality development of tourism.

### High-quality development of tourism

As China has entered a new development stage, the goal of tourism development has changed accordingly and the concept of high-quality tourism development should be redefined. As a result, interest in high-quality tourism development has grown since 2018, with a surge in the number of studies.

According to the 2030 Sustainable Development Goals (SDGs), sustainable development includes maintaining a high quality of development [8]. As a participating country in the sustainable development goals, China has adopted a development model driven by "innovation, coordination, greenness, openness, and sharing". These five major development concepts can be considered as an extension of the 2030 Agenda’s five pillars: people, planet, prosperity, peace, and partnership. According to the Quality Support Committee of the United Nations World Tourism Organization (UNWTO), high-quality tourism refers to a form of collaboration among tourism stakeholders in providing tourists with an exceptional experience while pursuing their own interests. One of the most important aspects of high-quality tourism is the efficient use of resources to achieve a balance between the human social system and the rest of the ecosystem to ensure humanity is living in harmony with nature [9]. Therefore, high-quality tourism development belongs to sustainable development. The main goal of this advanced type of development is to promote human development and meet all human needs without sacrifice the nature. Sustainable development requires that people must contribute to economic growth, social progress and promote environmental sustainability.

To ensure a sustainable tourism development in targeted regions, good governance should be integrated into multiple aspects [10]. Ethical entrepreneurship in tourism involves a variety of ethical behaviours to achieve goals with a notion of the greater good [11]. Ateljevic has a vision of connecting regenerative agriculture and transformative tourism to reset the global tourism system for good [12]. New tourism economic structures such as collaborative or sharing economy, circular economy, creative economy, gift economy, sacred economy and regenerative economy are becoming mature [13]. As a labor-intensive industry, tourism could be harnessed as a means to eliminate poverty and substantially boost the employment of impoverished people [14]. Improving workforce conditions in tourism can transform regional tourism into a sustainable, well-paid and well-educated year-round occupation with opportunities for long-term careers [15]. Building justice, equity and empowerment in communities engaged with tourism is essential to address the multiple crises of tourism, including injustice, oppression and marginalization [16].

As one of the sectors hit hardest by the COVID-19 pandemic, the tourism sector is facing new development challenges after COVID-19. In order for the tourist industry to recover, the tourism sector needs to adapt to new situations, strengthen its capacity to handle risks on its own. Instead of merely going back to the way things were, countries should shift to a more resilient, sustainable and inclusive models of tourism [17]. While tourism scholars’ dominant discourse and rhetoric views tourism as an “engine of economic growth and development”, a growing literature calls to re-examine the ontological assumptions underlying current economic thinking in the sustainability discussion in tourism [18]. Tourism should be reclaimed from an industry that has defined it as a business sector for their profit accumulation, to a human endeavour based on the rights and interests of local communities in welcoming tourists [19]. Transformative tourism research needs to challenge existing paradigms and critically evaluate its ontological and epistemological foundations, rethinking contemporary science, growth and technology paradigms [20].

Data analytics and smart technologies have played a crucial role in the post covid transformation of tourism. Business intelligence and big data are evolving fast, and AI applied to data mining and predictive learning is enhancing the intelligence capabilities of tourism and hospitality organizations and assisting them in understanding fast changing and competitive market [21]. Smart tourism destinations enable the "digital transformation" of destinations through smart tourism technologies to create a commercial and human value for visitors with a focus on sustainability, experiences and efficiency, thereby constructing a sustainable path of destination development [22]. This lays a foundation for the high-quality tourism development. Four criteria can be used to categorize the primary driving forces that affect the high-quality tourism development in China and they are consumption demand, capital investment, national policies, and technical innovation [23–26]. Moreover, institutional change can contribute to better economic outcomes and higher quality of tourism [27].

### Evaluation of the quality of tourism development

The quality indicator is a measure that assesses a particular process or outcome, and it can be related to the structure, process, outcome or sustainability. According to the study on the evaluation of tourism competitiveness, all the factors affecting the evaluation are related to market conditions, social development, environmental policies, human resource, infrastructure, and technical advancement [28]. To make the evaluation more efficient, scholars proposed an evaluation chart for sustainable tourism [29]. Previous studies have evaluated the tourism from perspectives of economy, society, environment, time, and quality [30–35]. Many scholars use the comprehensive indicator system to measure the quality of tourism development. For example, the SERVQUAL model is often used to figure out the service quality in the tourism sector [36,37]. Some scholars have examined the quality of tourism development based on efficiency, structure, and environment [27]. Others analyzed the quality and spatial differences of tours that cross provincial boundaries in China. Researchers established an evaluation index system with 31 indicators and 5 subsystems to evaluate the effects of coordinating “quality” with “quantity” in high-quality tourism development [38]. Kim et al. built an evaluation system that comprised 18 sub-domains and 60 items and identified 7 domains within the system: heritage and local identity, tourism and hospitality, quality of urban landscape, environment and energy, infrastructure, education, and conviviality [39].

In empirical studies, Shi et al. built a comprehensive evaluation index system with 40 indicators from six high-quality development dimensions, including resources, facilities, economy, environment, innovation, and the integration of culture tourism [40]. Based on the coordination between the high-quality development of tourism and the ecology, Long et al., created an index system that measured tourism quality from four perspectives: environment, resources, service quality, and the attractiveness of tourist destinations. The other 9 aspects and 26 indicators include pollution, ecological background, scenic resource, cultural resources, accommodation conditions, service capacity, service quality, tourism flows and tourism income [41]. Based on the five development concepts, Tang et al. proposed an index system based on dimensions of innovation, coordination, green, open, sharing, and effectiveness to study the high-quality tourism development in Hunan Province [42]. Yan et al. focused more on evaluating the economic vitality to reflect China’s high-quality tourism development, so they established an evaluation index system from seven dimensions: industry, innovation, coordination, green, open, sharing, and effectiveness. Using Entropy weight method (EWQI) and Geographic Information System (GIS), they conducted an empirical analysis on 30 provinces/cities in China [43]. Based on the concepts and requirements of high-quality tourism development, Liu et al. built an evaluation index system with 53 measurement indicators and from five dimensions: the supply and demand, innovation, ecological civilization, economic efficiency, and people’s quality of life [44].

In summary, although the evaluation of tourism competitiveness based on sustainable development has achieved fruitful results, the research that evaluates the quality of tourism development is still at early stage. In addition, most empirical studies are evaluations at the national level, and few of them have evaluated provincial areas, and the evaluation methods are not comprehensive enough, which reduces the validity of the studies. Therefore, this study builds an evaluation index system based on the concept of high-quality tourism development. It has selected panel data of 9 cities in Fujian Province, China, and uses the combination of the TOPSIS method based on entropy-AHP weights for empirical analysis of the influencing factors of high-quality tourism in different regions. It not only enhances the theory of high-quality tourism development, but also offers insights for the tourism development in specific regions.

## Data sources and research methods

This section introduces the methodologies used in the study of high-quality tourism development. Statistical analysis needs to adopt representative samples, so we selected some representative areas for further investigation [45]. The origins of significant data are explicitly identified and attributed. Finally, the detailed research procedures and steps will be shown.

### Overview of the study area

Nine prefecture-level cities in the Fujian Province were chosen by this study as research areas, including Fuzhou, Xiamen, Quanzhou, Putian, Ningde, Zhangzhou, Nanping, Longyan and Sanming. Fujian Province is chosen as the research object for studying the high-quality tourism development because it is representative in two aspects. First, tourism has long been a pillar of the Fujian Province’s economic fabric and Fujian has listed cultural tourism as one of the "four major components of its economic base". Growing travelers’ interest in cultural travel is a prominent factor driving the global market forward and helping China’s domestic tourism to fully recover. In the first quarter of 2023, the total number of tourists received by the province was 110.7958 million, recovering by 107.2% compared with the same period in 2019. The province generated 132.28 billion yuan in total tourism revenue, recovering by 88.5% compared with the same period in 2019. Second, Fujian Province is rich in tourism resources and has prominent regional and geographical advantages. Fujian province had the highest forest coverage in China, reaching approximately 66.8 percent of the land area. Fujian is rich in cultural heritage resources, with 9 projects included in the UNESCO’s Intangible Cultural Heritage list (roster) and it has become the first province in China that has intangible heritage on UNESCO’s lists of the Intangible Cultural Heritage of Humanity, Intangible Cultural Heritage in Need of Urgent Safeguarding and the Register of Good Safeguarding Practices. In addition, there are 167 world heritage sites, national geoparks, national parks, national nature reserves, national forest parks, national wetland parks, cultural relics, etc. Destinations including Fuzhou, Pingtan, Wuyi Mountain, Xiamen, and Dongshan are visited by tourists from all over the world.

### Data source

The data in this study is from *China City Statistical Yearbook* (2015–2021), *Fujian Statistical Yearbook (2015–2021)*, and the *Statistical Bulletin on National Economic and Social Development of Fujian Province (2015 to 2021)* released by Fujian Provincial People’s Government, and the database of related municipal tourism bureau.

### Research process

There are two main methods to determine the weight of indicators. The first type is subjective weighting methods, which include the analytic hierarchy process method (AHP), cluster analysis method, and principal component analysis method (PCA), etc. When adopting weights to reflect reality or ideal situation, researchers may subjectively determine the weights based on their own preference or experience. The second type are objective weighting methods, which include entropy analysis method and Vlse Kriterijumska Optimizacija Kompromisno Resenje analysis method (VIKOR). Researchers use numerical calculation to avoid bias in the subjective weighting methods and reflect the real status of multidimensional indicators. However, the differences between indicators, when being added together, may increase the weight error and affect the final results [46]. The TOPSIS model is a multi-objective decision-making technique that helps determine the optimal ranking [47]. The advantages and disadvantages of different evaluation objects are determined by their distance to the positive and negative ideal solutions. The best solution has the shortest distance from the positive-ideal solution, and the longest distance from the negative-ideal one. TOPSIS model should be first established and then combine this model with the entropy and AHP weights. In this way, the distance between objects and positive and negative best solutions can be effectively measured, evaluated, and ranked. The advantages of the methods in this paper include: (1)they are simple to calculate; (2) they highlight the relative significance of different indicators, making the relative change clearer; (3) they avoid the drawbacks of relying solely on one method to make sure the results are objective;(4) they have been used in other research domains [48].

The main goal of data processing is to standardize each quantitative index’s original data in order to eliminate dimensional differences. The objective weight and the information entropy value are calculated using the entropy weight method, while the subjective weight is calculated using the AHP method. The close degree of progress (CDP) and levels of high-quality tourism development in cities of Fujian Province are evaluated using TOPSIS method. The specific steps are as follows:

#### Index weight

First, index variables that can describe the problem and the original data of samples are collected, and standardized processing is performed as follows:

$$G_{ij}=\frac{x_{ij}}{\sum_{i=1}^{m}x_{ij}},$$ (2.1)

where *x*_*ij*_ is the original data of the *i*−th sample and *j*−th index, *G*_*ij*_ is the normalization result of *x*_*ij*_, *i* = 1,2,…,*m*; *j* = 1,2,…*n*.

### Calculation of information entropy

$$Z_{j}=-\frac{\sum_{i=1}^{m}G_{ij}\;\ln\;G_{ij}}{\ln\;m},$$ (2.2)

Where *m* is the number of indexes, *j* = 1,2,…*n*.

### Calculation of objective weight ${\hat{W}}_{j}$(Entropy weight, denoted by EW)


$${\hat{W}}_{j}=\frac{1-Z_{j}}{n-\sum_{j=1}^{n}Z_{j}},\;j=1,2,\ldots n,$$ (2.3)

AHP is used to obtain the subjective weight ${\bar{W}}_{j}$. By comparing the relative importance of the indicators, AHP method determines the weight of the index layer to the overall objective of evaluation. Pairwise comparison is used to determine the relative influence of the evaluation index on high-quality tourism development. Eight experts were invited to compare and evaluate the high-quality tourism development in cities of Fujian Province, including researchers from Fuzhou University, Fujian Jiangxia University, Fuzhou University of International Studies and Trade, and officials from Fujian Provincial Department of Culture and Tourism who oversee and manage the tourism development of the province. The experts used Python 3.9 software to calculate each indicator’s relevance and assigned a score. After the consistency test was passed, the indicator’s weight could be determined.

#### Calculation of the combination weight (CW)

To make up for the shortcomings of subjective and objective weights, the fourth step is to get the combination weight. Thus, the combination weight of objective weight ${\hat{W}}_{j}$ and subjective weigh ${\bar{W}}_{j}$ is obtained:

$$W_{j}=\frac{\sqrt{{\hat{W}}_{j}{\bar{W}}_{j}}}{\sum_{j=1}^{n}\sqrt{{\hat{W}}_{j}{\bar{W}}_{j}}},\;j=1,2,\ldots n,$$ (2.4)

#### Close degree of progress (CDP) based on TOPSIS method

To assess the relative merits and demerits of each research object, the TOPSIS method is used to calculate the distance between the evaluation object and the positive and negative best solution. A hypothetical solution (scheme) is optimal if it is closest to the positive best solution and far away from the negative best solution. The details are introduced as follows:

- The first step is to use the extremum method value to process the original data and get the decision matrix *K* = [*k*_*ij*_]:
$$k_{ij}=\frac{x_{ij}-\min_{i,j}\{x_{ij}\}}{\max_{i,j}\{x_{ij}\}‐\min_{i,j}\{x_{ij}\}},\;i=1,2,\ldots m;j=1,2,\ldots n.$$ (2.5)
- The second step is to construct the weighted decision matrix:
$$P_{m\times n}={\left(p_{ij}\right)}_{m\times n}=(\begin{array}{cccc} w_{1}k_{11} & w_{2}k_{12} & \ldots & w_{n}k_{1n}\\ w_{2}k_{21} & w_{2}k_{22} & \ldots & w_{n}k_{2n}\\ \vdots & \vdots & \ldots & \vdots \\ w_{1}k_{m1} & w_{2}k_{m2} & \ldots & w_{n}k_{mn} \end{array}).$$ (2.6)
  Where wj represents the weight of comprehensive indicators from Formula (2.4), and *k*_*ij*_ denotes the weight of normalization from Formula (2.6), *i* = 1,2,…,*m*; *j* = 1,2,…*n*.
- Calculation of the solutions of positive ideal and negative ideal:
$$P^{+}=\{\max\;p_{ij}|i=1,2,\cdots ,m\}=\{p_{1}^{+},p_{2}^{+},\cdots ,p_{m}^{+}\},$$ (2.7)
$$P^{-}=\{\min\;p_{ij}|i=1,2,\cdots ,m\}=\{p_{1}^{-},p_{2}^{-},\cdots ,p_{m}^{-}\}.$$ (2.8)
- Calculation of the close degree of progress (CDP):
$$F_{i}=\frac{\sqrt{\sum_{j=1}^{n}{\left(p_{j}^{-}-p_{ij}\right)}^{2}}}{\sqrt{\sum_{j=1}^{n}{\left(p_{j}^{+}-p_{ij}\right)}^{2}}+\sqrt{\sum_{j=1}^{n}{\left(p_{j}^{-}-p_{ij}\right)}^{2}}}.$$ (2.9)
  Where *p*^+^ and *p*^-^ indicates the maximum and minimum values of the i-th low of the weighted decision matrix *P*_*m*×*n*_, respectively, and *p*_*ij*_ indicates the elements of weighted decision matrix *P*_*m*×*n*_, *i* = 1,2,…,*m*; *j* = 1,2,…*n*.
  The Formula (2.9) is used in this study to determine the CDP of 9 cities in Fujian Province, based on the previously mentioned processes. [0,1] is the range of values and larger value is closer to the optimal solution.

## Construction of influence factors and index system

### Definition of high-quality development of tourism

The tourism industry is a vital part of the economy. High-quality tourism growth will increase material wealth by efficiently allocating tourism resources and upgrading industrial structure through innovative technologies and the open market. Regions with high-quality tourism will reach a balance between tapping their tourism resources and preserving environment, and share resources with other regions in the tourism industry. Tourism can generate quality employment opportunities for durable economic and social growth. As long as it is well-organized and sustainable, high-quality tourism development will create a green and virtuous circle to help residents live in harmony with nature and enjoy a happy life. Meanwhile, high-quality tourism development cannot be limited, we need to be open and accept the economies and cultures of other regions with an open mind and inclusive attitude, and we should share and learn from each other. A high-quality tourism should be distinctive and inclusive, with higher influence to local areas and more benefits to local people. Innovation and the cultivation of professional talents are also driving forces for growth. Moreover, high-quality development of tourism is not only the development of the tourism sector itself, it is a comprehensive development related to science, technology, and innovation and it includes industrial synergy, green development, and open sharing. Tourism development prioritizes efficiency over speed, and pays more attention to the development quality and sustainability. It focuses on tourism-based economic growth that contributes to the economy, society, and the environment all at the same time.

### Element model of high-quality development of tourism

**Fig 1:**
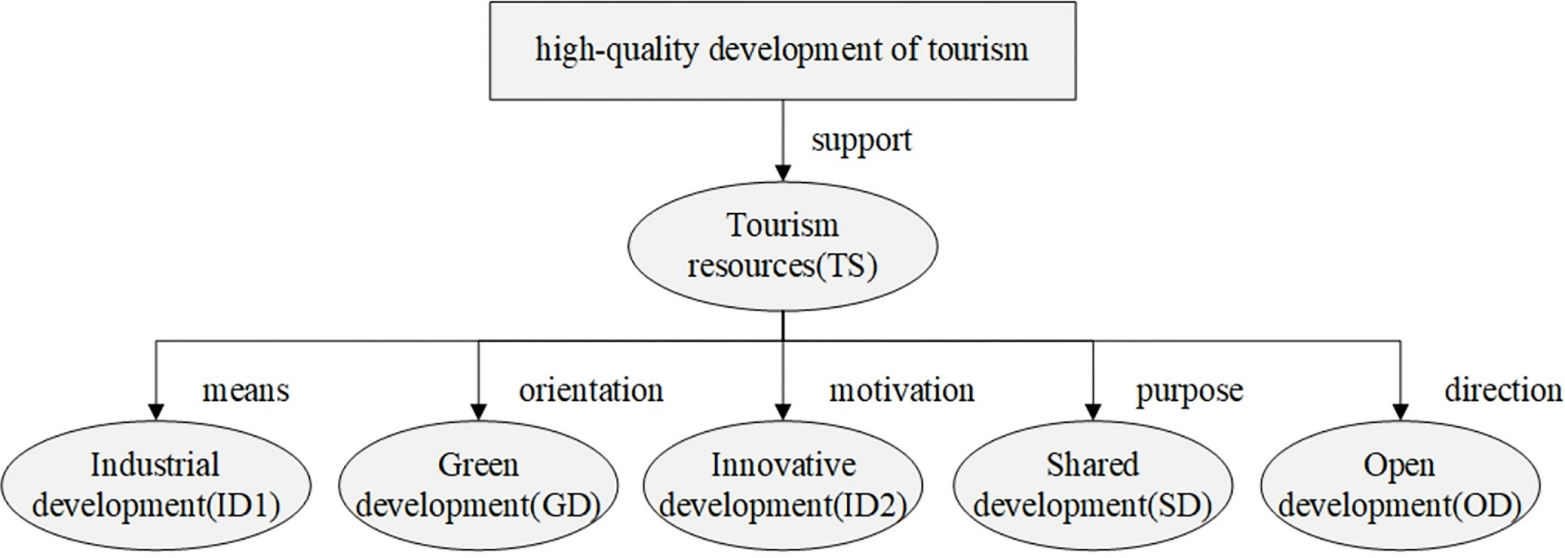
Element model of high-quality development of tourism.

Based on the definition of high-quality development of tourism and results of previous research [4], this paper has developed an element model to reflect high-quality tourism development (Fig 1). To meet people’s growing demand for high-quality tourism and leisure, we should manage tourism development based on quality of visitation, not quantity of visitors. Moreover, we need to follow the new concept of tourism development, deepen the supply-side structural reform and address imbalance problems in tourism supply. The high-quality development of tourism depends on resources as a base for growth, coordination to help the growth of industrial connections, going green to support sustainable development, innovation to encourage efficient and diversified development, sharing to promote peaceful growth, and openness to foster cooperation.

**Tourism resources (TS)** Tourism resources are the natural and anthropogenic elements that attract tourists to a destination. These resources are crucial for the high-quality development of tourism. Traditional tourism resources mainly include scenery and cultural landscape [49]. Modern tourism resources show more diversity. In addition to the landscape resources, modern tourism also includes history and humanities, urban and rural landscape, modern artificial landscape, such as the Olympics venues, exhibitions, monuments, and other important historical events. These resources, whether they are used alone or together, create more alternatives for tourism development. How to fully use both traditional and modern tourism resources becomes an important topic. They are also important indicators to evaluate the quality tourism growth.

**Industrial development (ID1)** Industrial development is an important economic factor for the high-quality development of tourism. The coordinated development of high-quality tourism not only creates jobs in the tertiary sector, such as accommodation, catering, transportation, but also encourages growth in the primary and secondary sectors of industry [50]. At current stage, the sector is still challenged by unbalanced growth and inadequate development. There are problems such as a limited form of industrial integration, weak policy framework, an outdated tourism master plan, and obvious regional gap. The tourism products offering cannot match the demand of tourists, and there is still a long way ahead if we want to reach the goals of high-quality development. Consequently, the high-quality development of the tourism sector depends on the optimization of industrial structure, the balanced development of supply and demand, and the integration of industries [12]. To have a high-level structure of the tourism sector, it is crucial to adjust regional industrial structure, expand the industrial chain, promote innovation in industrial clusters, reform the supply-side structure, and boost consumption.

**Green development (GD)** Green development is important for the high-quality and sustainable development of tourism. As one of the world’s biggest and fastest growing economies, China has laid out targets to mitigate climate change, with its so-called "dual carbon" goals, which aims to peak carbon dioxide emissions by 2030 and achieve carbon neutrality before 2060. The tourism industry relies heavily on the proper conservation and sustainable management of the environment. During the development of tourism, the extensive development model in the past has caused some irreversible ecological damages in some areas. To make sure the tourism grows in a green and high-quality way, we need to put more efforts in “consolidation”. For example, when managing regional resources, we should take into account the environment’s carrying capacity and use technologies to manage tourism resources and carry out tourism environment governances. The second is “green innovations”, by encouraging eco-friendly accommodations, purchase and use of products with lower environmental impacts, and travels that use low emissions means of transport. We need to integrate ecological civilization and green development into every part of high-quality tourism development [51].

**Innovative development (ID2)** High-quality tourism development is also driven by innovative development, which is also a key to improving tourism competitiveness. Innovation can be made from a variety of dimensions, such as resources, industries, environmental protection, and sharing economy. The basic idea behind high-quality and innovative tourism development is that it includes new ways to do tourism, such as adopting business intelligence, big data, and smart tourism technologies to provide tourism services, management, marketing, and experience [21]. For example, smart tourism is a new buzzword applied to describe the increasing reliance of tourism destinations [22]. In the past decade, smart tourism has played an important role in promoting the tourism economy, contributing to the tourism experience and the risk management during the epidemic [52]. However, the development of smart tourism varies in different regions due to differences in regional policies, resources, and talents. It should be further strengthened and the development of tourism in the future must be driven by innovation.

**Shared development (SD)** According to “The 14th Five-year Plan”, the benefits of tourism growth should be shared with the people and tourism should act as a positive force to serve and help people. A high-quality and shared development of tourism needs both the “hosts” and the “customers” to feel satisfied, happy, and safe during the consumption experience. Measures should be adopted to improve facility management, balance product supply and customer demand, and enhance market supervision. The ultimate goal is to make sure that the leisure travel is “safe, orderly, high-quality and efficient”. Looking from the definition of the shared development, the comprehensive sharing of tourism development means the share of resources, policies, culture and brand, services, market and space [53]. Moreover, regional policies and mechanism, medical and health services, cultural resources, and tourism platforms are a few examples to be shared. The sharing economy enables the sustainable development of tourism and at the same time contributes to social well-being, in which local people are involved in high-quality tourism development and enjoy its benefits [14].

**Open development (OD)** China has adopted both “dual cycle” development strategy and the “bring in” and "go global" strategy. These plans will help the high-quality tourism development find a balance between domestic economy and global economy so that it will be open, inclusive, balanced, and win-win. The focus of the international tourism development strategy overlaps with the goal of high-quality open development. This includes revitalizing inbound and outbound tourism and promoting cross-cultural communication. During the process, green development and crisis management should be paid attention to.

### Evaluation index system for high-quality tourism development

The construction of the evaluation index system is rooted in established models, with the selection of evaluation indicators guided by the following principles [54]. First, the indices should align with the national strategy and policies for high-quality tourism development. Second, they should reflect the factor model of high-quality tourism development. Third, the indicators should enjoy widespread acceptance and citation. Fourth, the indicators should be straightforward and unambiguous. Fifth, the data for these indicators should be readily obtainable.

Drawing from the high-quality tourism development factor model, this paper draws inspiration from the research findings in related domains to formulate an evaluation index system for tourism. This system encompasses six key dimensions: tourism resources, industrial development, green sustainability, innovation, inclusiveness, and international openness. The index system consists of one primary indicator and six secondary indicators. For the sake of data representativeness and accessibility, 24 tertiary indicators are selected to facilitate the evaluation and analysis of high-quality tourism development across various dimensions and overall developmental stages. All indicators are positively oriented, as delineated in Table 1.

**Table 1:** Evaluation index system and weight.

Level 1	Level 2	Level 3	EW	AHP	CW	Rank
		Number of 5A Scenic spots	0.0599	0.0179	0.0313	14
	Tourism	Number of cultural performance groups	0.0095	0.0318	0.0417	10
	Resources	Number of Museums	0.0292	0.0521	0.0533	7
	(0.1086)	Number of books in library	0.0417	0.0069	0.0194	21
		Turnover of tourism-related industries	0.0524	0.0288	0.0397	11
	Industrial	Number of beds in accommodation industry	0.0238	0.0155	0.0291	16
	Development	Financial expenditure of tourism-related industries	0.0278	0.0093	0.0226	19
	(0.1086)	Tourist reception	0.0117	0.0551	0.0548	6
		Number of national nature reserves	0.047	0.0593	0.0569	5
High-	Green	Green area of park	0.0469	0.0242	0.0364	12
quality	Development	Environmental financial expenditure	0.0077	0.1607	0.0937	1
develo-	(0.4049)	Harmless treatment rate of household garbage	0.0021	0.1607	0.0937	1
pment		Local finance expenditure on science and technology	0.0383	0.0454	0.0498	8
of	Innovation	The number of tourism majors offered by universities	0.0571	0.1323	0.0851	3
tourism	Development	Number of mobile Internet users	0.0191	0.0808	0.0664	4
	(0.2761)	Number of technical personnel in accommodation and catering industry	0.0779	0.0175	0.0311	15
		People engaged in tourism-related industries	0.0463	0.0081	0.0209	20
	Shared	Regional security incident reduction rate	0.0555	0.0146	0.0282	17
	Development	Number of health facilities per 10,000 tourists	0.0265	0.0357	0.0442	9
	(0.0616)	Tourism revenue	0.0224	0.0033	0.0134	24
		Foreign exchange income from international tourism	0.0719	0.0041	0.0148	22
	Open	Proportion of tourism foreign exchange income in total tourism income	0.0439	0.0115	0.0251	18
	Development	Number of foreign-funded tourist hotels	0.1027	0.0209	0.033	13
	(0.0402)	Visitor arrivals	0.0871	0.0041	0.0148	22

Within these dimensions, the tourism resources dimension, as inspired by Long et al.’s work [41], leverages the Number of 5A-rated tourist attractions to represent traditional tourism resources, evaluating the development of high-quality scenic attractions. Additionally, Number of cultural performance groups, Number of Museums, Volume of books in library, representing modern tourism resources, gauge the region’s contemporary cultural and tourism resources, including recreational, historical, cultural and knowledge-based assets, along with their sharing potential. These four indicators holistically assess the region’s capacity for unearthing and expanding its cultural and tourism resources.

In the remaining dimensions, the selection of tertiary indicators adheres to the evaluation index system for high-quality tourism development established by Tang et al. [42] and Yan et al. [43], and follows the input-output evaluation paradigm. In the dimension of industrial development, the focus centers on evaluating the extent of synergistic development between tourism and other related industries, such as accommodation, catering and transportation. Turnover of tourism-related industries and Number of beds in the accommodation industry signify the governmental and industrial investment in these related sectors, respectively. The effect of synergistic development among tourism-related industries is assessed through Financial expenditure in tourism-related industries and Tourist reception.

To evaluate the green financial input of the region, Environmental financial expenditure is employed as proxy. Additionally, indicators such as Number of national nature reserves, Green area of parks, and Harmless treatment rate of household garbage were utilized to evaluate the region’s green and high-quality development in terms of green tourism resources and environmental governance accomplishments.

In the innovation development dimension, the focus is on assessing the input-output dynamics of technology empowerment. Local finance expenditure on science and technology, Number of technical personnel in the accommodation and catering industry, and Number of tourism majors offered by universities reflect the financial investment in science and technology, human resources in science and technology, and the region’s innovation *p*otential. Furthermore, Number of mobile Internet users serves as the foundation for smart tourism development.

The dimension of inclusivity assesses tourism’s to regional safety environment, medical and healthcare support, and the overall coverage of tourism development benefits. Regional security incident reduction rate and the Number of health facilities per 10,000 tourists evaluate the safety environment and healthcare support, while People engaged in tourism-related industries and Tourism revenue gauge the extent of tourism development’s reach.

For open development, indicators such as Foreign exchange income from international tourism, Proportion of tourism foreign exchange income in total tourism income, and Visitor arrivals are employed to evaluate the regional tourism’s international influence, economic contributions, and open development achievements. The Number of foreign-funded tourist hotels reflects the level of support for open development.

## Results and analysis

The research methods outlined in Section 2 were utilized to calculate the close degree of progress in high-quality tourism development in 9 cities within Fujian Province from 2016 to 2020. Data were analyzed with a focus on both temporal trends and spatial disparities.

### Comprehensive analysis based on temporal and spatial development

#### Analysis of temporal development

**Fig 2:**
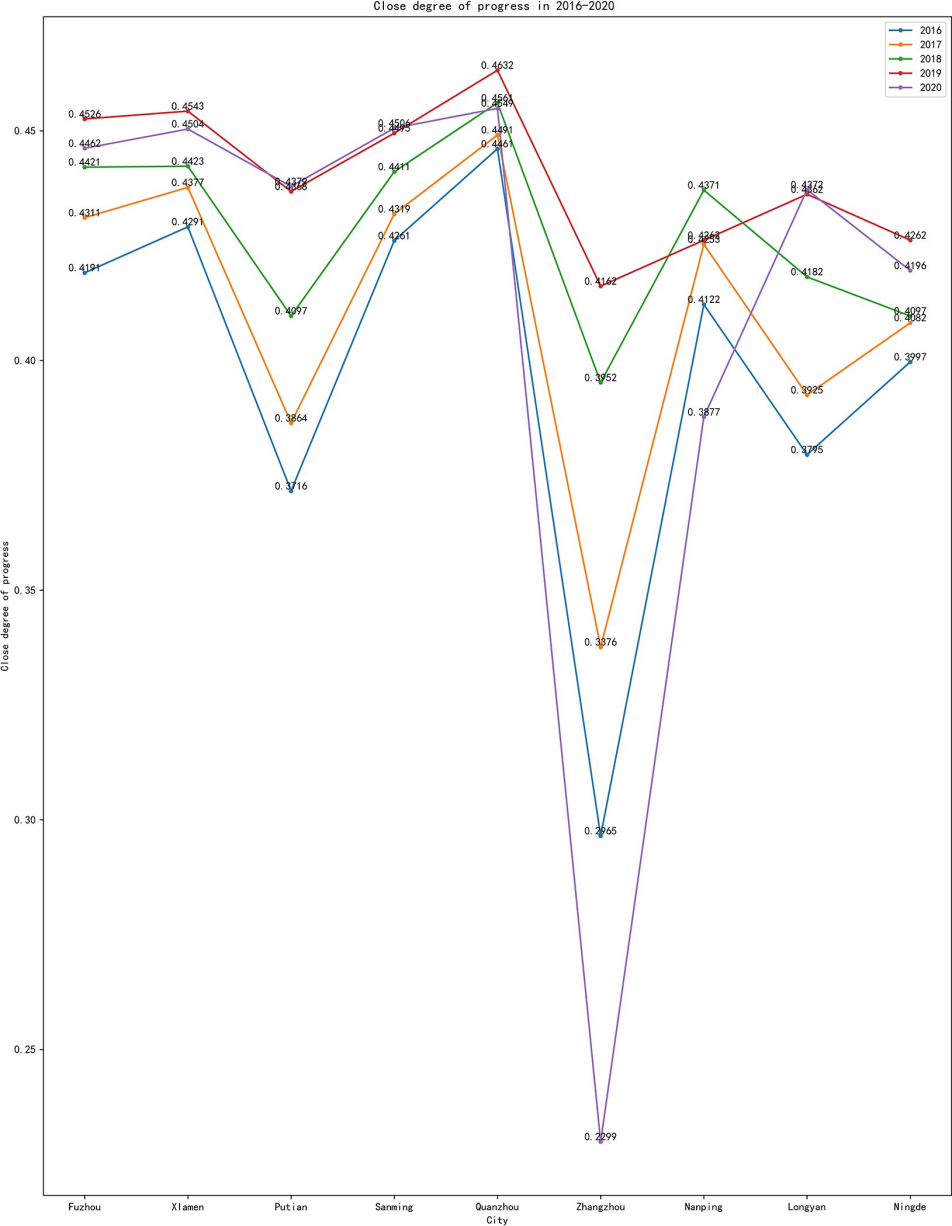
Close Degree of Progress (CDP) (time sequence) in 2016–2020.

A time series evolution analysis, as depicted in Table 2 and Fig 2, reveals a consistent overall development trend in the 9 cities of Fujian Province. This trend reflects a steady upward trajectory from 2016 to 2019, characterized by commendable development efficiency and effectiveness. Notably, Fuzhou, Xiamen, and Quanzhou exhibited high overall progress levels with stable growth, maintaining annual growth rates between 2.5%-3%. In the years 2018 to 2019, certain areas experienced accelerated growth rates, with Putian, Zhangzhou, Longyan, and Ningde registering increases of 6.61%, 5.31%, 4.3%, and 4.02%, respectively.

**Table 2:** Close Degree of Progress (CDP) (time sequence).

City	Year	TR	ID1	GD	ID2	SD	OD	Whole CDP
	2016	0.4371	0.4173	0.4386	0.2982	0.4661	0.3835	0.4191
	2017	0.4296	0.4321	0.4436	0.3089	0.4674	0.4136	0.4311
Fuzhou	2018	0.4491	0.4451	0.4507	0.3836	0.4701	0.4545	0.4421
	2019	0.4561	0.4572	0.4588	0.4395	0.4752	0.4621	0.4526
	2020	0.4614	0.4491	0.4694	0.4382	0.4739	0.2688	0.4462
	2016	0.4433	0.4322	0.4434	.3704	0.2981	0.4195	0.4291
	2017	0.4446	0.4426	0.4148	0.3831	0.2987	0.4298	0.4377
Xiamen	2018	0.4496	0.4479	0.4472	0.3943	0.3131	0.4468	0.4423
	2019	0.4548	0.4622	0.4623	0.4147	0.4395	0.4537	0.4543
	2020	0.4651	0.4534	0.4641	0.4469	0.3301	0.1964	0.4504
	2016	0.4069	0.3541	0.4424	0.4587	0.4851	0.3693	0.3716
	2017	0.4115	0.3738	0.4053	0.4621	0.4865	0.4532	0.3864
Putian	2018	0.4649	0.4055	0.4153	0.4702	0.4868	0.4387	0.4097
	2019	0.4692	0.4438	0.4074	0.4711	0.4866	0.4717	0.4368
	2020	0.4768	0.4462	0.4641	0.4579	0.4842	0.1482	0.4379
	2016	0.4764	0.4178	0.4526	0.4468	0.4643	0.3555	0.4261
	2017	0.4678	0.4273	0.4578	0.4474	0.4652	0.4485	0.4319
Sanming	2018	0.4691	0.4415	0.4372	0.4504	0.4668	0.4837	0.4411
	2019	0.4703	0.4574	0.4311	0.4225	0.4693	0.5569	0.4495
	2020	0.4715	0.4608	0.4617	0.3906	0.4749	0.3011	0.4506
	2016	0.4021	0.4393	0.4792	0.4651	0.4825	0.4594	0.4461
	2017	0.4254	0.4434	0.4779	0.4678	0.4828	0.3662	0.4491
Quanzhou	2018	0.3933	0.4535	0.4812	0.4725	0.4844	0.4117	0.4561
	2019	0.4159	0.4646	0.4809	0.4746	0.4855	0.4101	0.4632
	2020	0.4586	0.4511	0.4906	0.4746	0.4835	0.1471	0.4549
	2016	0.4853	0.2791	0.4726	0.4091	0.4053	0.3943	0.2965
	2017	0.4864	0.3361	0.4729	0.4096	0.4164	0.4236	0.3376
Zhangzhou	2018	0.4875	0.4076	0.4732	0.4544	0.4275	0.448	0.3952
	2019	0.4863	0.4381	0.4733	0.4178	0.4466	0.4733	0.4162
	2020	0.4875	0.1728	0.4802	0.4262	0.3625	0.2274	0.2299
	2016	0.4838	0.4191	0.4424	0.3724	0.4637	0.3623	0.4122
	2017	0.4841	0.4386	0.4533	0.3769	0.4643	0.4878	0.4253
Nanping	2018	0.4842	0.4505	0.3703	0.4288	0.4656	0.3045	0.4371
	2019	0.4851	0.4271	0.3745	0.4473	0.4581	0.3129	0.4262
	2020	0.4864	0.3505	0.4041	0.4384	0.4415	0.0763	0.3877
	2016	0.4669	0.3784	0.4514	.3383	0.4844	0.3587	0.3795
	2017	0.4766	0.3961	0.4574	0.3417	0.4847	0.3947	0.3925
Longyan	2018	0.4929	0.4262	.4102	0.3846	0.4857	0.4311	0.4182
	2019	0.4976	0.4499	0.4121	0.3926	0.4868	0.4748	0.4362
	2020	0.4831	0.4432	0.4145	0.4428	0.4866	0.1966	0.4372
	2016	0.4647	0.4475	0.4609	0.1366	0.4769	0.3677	0.3997
	2017	0.4761	0.4546	0.4451	0.1542	0.4776	0.3777	0.4082
Ningde	2018	0.4907	0.4547	0.4332	0.1856	0.4778	0.3902	0.4097
	2019	0.4901	0.4581	0.4319	0.3596	0.4801	0.4705	0.4262
	2020	0.4792	0.4131	0.4649	0.4367	0.4733	0.3523	0.4196

However, Nanping region exhibited fluctuations, primarily influenced by the levels of industrial and shared development. In 2020, all cities witnessed varying degrees of decline, mainly due to the profound impact of COVID-19, leading to a sharp drop in inbound tourism and tourist foreign exchange income. Notably, Zhangzhou experienced the most significant decline, at 44.7%. In contrast, cities like Fuzhou, Xiamen, Nanping, and Ningde faced smaller declines of 0.2% to 1.5%, indicating their solid foundation for high-quality tourism development, which made them less susceptible to individual external factors. Putian and Longyan showed slight improvements in the Close Degree of Progress (CDP) in 2020, mainly attributed to advancements in green development and industrial innovation.

In summary, several cities along the southeast coast of Fujian are blessed with abundant tourism resources, a high degree of openness, a robust industrial base, and strong innovation potential, granting them a distinct advantage in high-quality tourism development. Nevertheless, the overall level of comprehensive tourism development varies slightly among individual coastal cities. In contrast, central, western, and northern Fujian boasts abundant ecological resources and have a strong base foundation in green development. Still, their levels of industry, innovation, and openness lag slightly behind those of southeast coastal Fujian, especially in the years 2016 and 2017. Thus, optimizing regional tourism development patterns, resource integration, and reducing regional disparities are pressing issues that must be addressed to enhance the overall level of high-quality tourism development in Fujian Province.

#### Analysis of spatial development

The comprehensive CDP based on spatial disparities, as presented in Table 3, highlights a noticeable imbalance in the overall high-quality tourism development across Fujian Province. This spatial development imbalance was particularly pronounced in 2016 and 2017. From 2018 to 2019, the development disparities among regions gradually diminished, and there was a significant increase in the levels of green development, innovative development, and shared development across all regions during the two years.

**Table 3:** Close Degree of Progress (CDP) (spatial differences).

Year	City	TR	ID1	GD	ID2	SD	OD	Whole CDP
	Fuzhou	0.4713	0.4433	0.4356	0.3102	0.4299	0.2312	0.4104
	Xiamen	0.3555	0.3872	0.4913	0.4386	0.4378	0.4901	0.3645
	Putian	0.0661	0.0896	0.2686	0.1057	0.1251	0.0797	0.0932
	Sanming	0.2325	0.0928	0.2845	0.1574	0.1136	0.0153	0.0997
2016	Quanzhou	0.1676	0.2225	0.2755	0.3591	0.2739	0.3854	0.2231
	Zhangzhou	0.1415	0.1288	0.2548	0.1583	0.1574	0.1105	0.1267
	Nanping	0.1591	0.0853	0.2147	0.0897	0.1255	0.1088	0.0908
	Longyan	0.1134	0.1231	0.3205	0.1897	0.1514	0.0286	0.1237
	Ningde	0.1102	0.1051	0.2394	0.0796	0.1268	0.0142	0.1036
	Fuzhou	0.4753	0.4428	0.4603	0.3057	0.4346	0.2377	0.4101
	Xiamen	0.3853	0.3781	0.4482	0.4382	0.4378	0.4812	0.3565
	Putian	0.0756	0.0876	0.2367	0.1088	0.1297	0.0991	0.0904
2017	Sanming	0.2147	0.0879	0.3149	0.1481	0.1157	0.0195	0.0941
	Quanzhou	0.2121	0.1991	0.2854	0.3437	0.2748	0.2469	0.1999
	Zhangzhou	0.1603	0.1362	0.2707	0.1489	0.1673	0.1175	0.1305
	Nanping	0.1762	0.0818	0.2373	0.0942	0.1264	0.1571	0.0871
	Longyan	0.1356	0.1211	0.3536	0.1793	0.1532	0.0301	0.1197
	Ningde	0.1351	0.0975	0.2229	0.0927	0.1291	0.0147	0.0974
	Fuzhou	0.4681	0.4426	0.4297	0.4055	0.4221	0.2668	0.4111
	Xiamen	0.3422	0.3584	0.4672	0.4376	0.4381	0.4729	0.3397
	Putian	0.0809	0.0885	0.2211	0.1249	0.1166	0.0827	0.0898
2018	Sanming	0.1751	0.0851	0.2297	0.1509	0.1044	0.0203	0.0901
	Quanzhou	0.1361	0.1965	0.2637	0.3469	0.2578	0.2685	0.1963
	Zhangzhou	0.1285	0.1556	0.2412	0.2121	0.1572	0.1202	0.1491
	Nanping	0.1425	0.0761	0.1431	0.1241	0.1135	0.0696	0.0833
	Longyan	0.1208	0.1287	0.2209	0.2279	0.1394	0.0315	0.1271
	Ningde	0.1211	0.0872	0.1786	0.1153	0.1153	0.0151	0.0893
	Fuzhou	0.4652	0.4435	0.4309	0.4376	0.2451	0.2652	0.4114
	Xiamen	0.3405	0.3706	0.4795	0.4347	0.4394	0.4714	0.3481
	Putian	0.0779	0.0852	0.1932	0.1176	0.0506	0.0921	0.0802
	Sanming	0.1659	0.0743	0.1965	0.1206	0.0469	0.0208	0.0747
2019	Quanzhou	0.1508	0.1944	0.2383	0.3134	0.1134	0.2574	0.1891
	Zhangzhou	0.1176	0.1521	0.2196	0.1421	0.0756	0.1318	0.1382
	Nanping	0.1356	0.0545	0.1309	0.1239	0.0472	0.0671	0.0633
	Longyan	0.1164	0.1271	0.2061	0.2058	0.0624	0.0371	0.1199
	Ningde	0.1114	0.0703	0.1601	0.1702	0.0518	0.0208	0.0758
	Fuzhou	0.4632	0.4452	0.4531	0.3849	0.4138	0.4061	0.4094
	Xiamen	0.3659	0.3675	0.4763	0.4386	0.4385	0.5022	0.3473
	Putian	0.0782	0.0829	0.2616	0.0698	0.0893	0.1017	0.0708
	Sanming	0.1616	0.0708	0.2667	0.0702	0.0915	0.0389	0.0652
2020	Quanzhou	0.1861	0.1733	0.2748	0.2453	0.2163	0.2971	0.1653
	Zhangzhou	0.1145	0.0409	0.2473	0.1058	0.0925	0.1614	0.0517
	Nanping	0.1351	0.0327	0.1628	0.0804	0.0783	0.0583	0.0447
	Longyan	0.1022	0.1236	0.2153	0.2131	0.1194	0.0481	0.1191
	Ningde	0.0992	0.0451	0.2133	0.1692	0.0879	0.0372	0.0605

**Fig 3:**
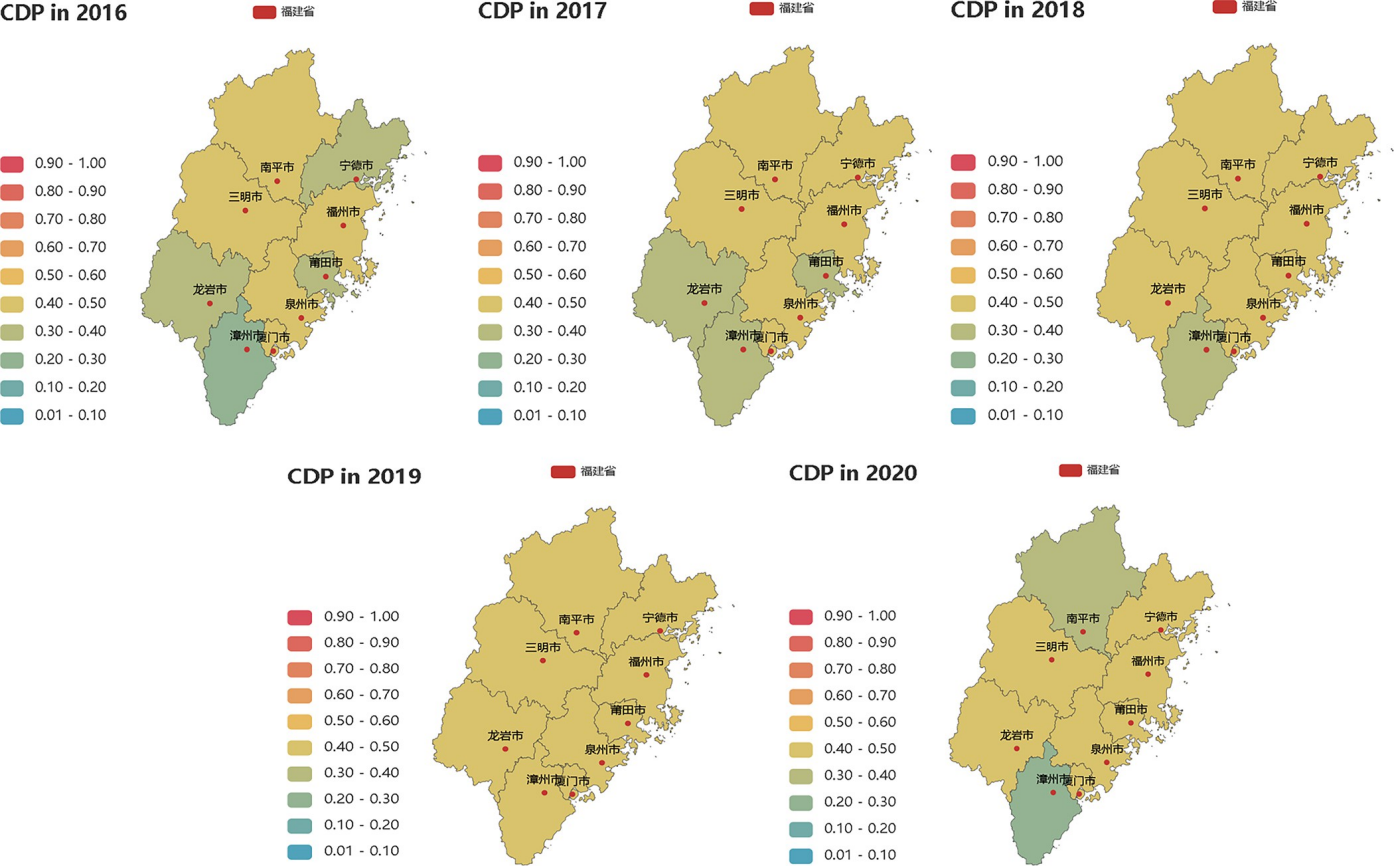
Close Degree of Progress (CDP) (spatial differences) in 2016–2020.

Over the period spanning 2016 to 2020, Fuzhou and Xiamen emerged as the frontrunners in high-quality tourism development, with comprehensive CDP levels of 0.4105 and 0.3512, respectively. Quanzhou ranked third with an average CDP of 0.1947, although it displayed some distance from the top two cities. The CDP levels of other cities ranged between 0.1192 and 0.0738. Notably, Zhangzhou and Nanping experienced considerable fluctuations in the comprehensive development from 2016 to 2020, largely attributed to the pandemic, and both cities demonstrated overall development levels that were below the desired mark (Fig 3).

### Analysis of various dimensions based on spatial differences

#### Analysis of tourism resources (Fig 4A)

**Fig 4:**
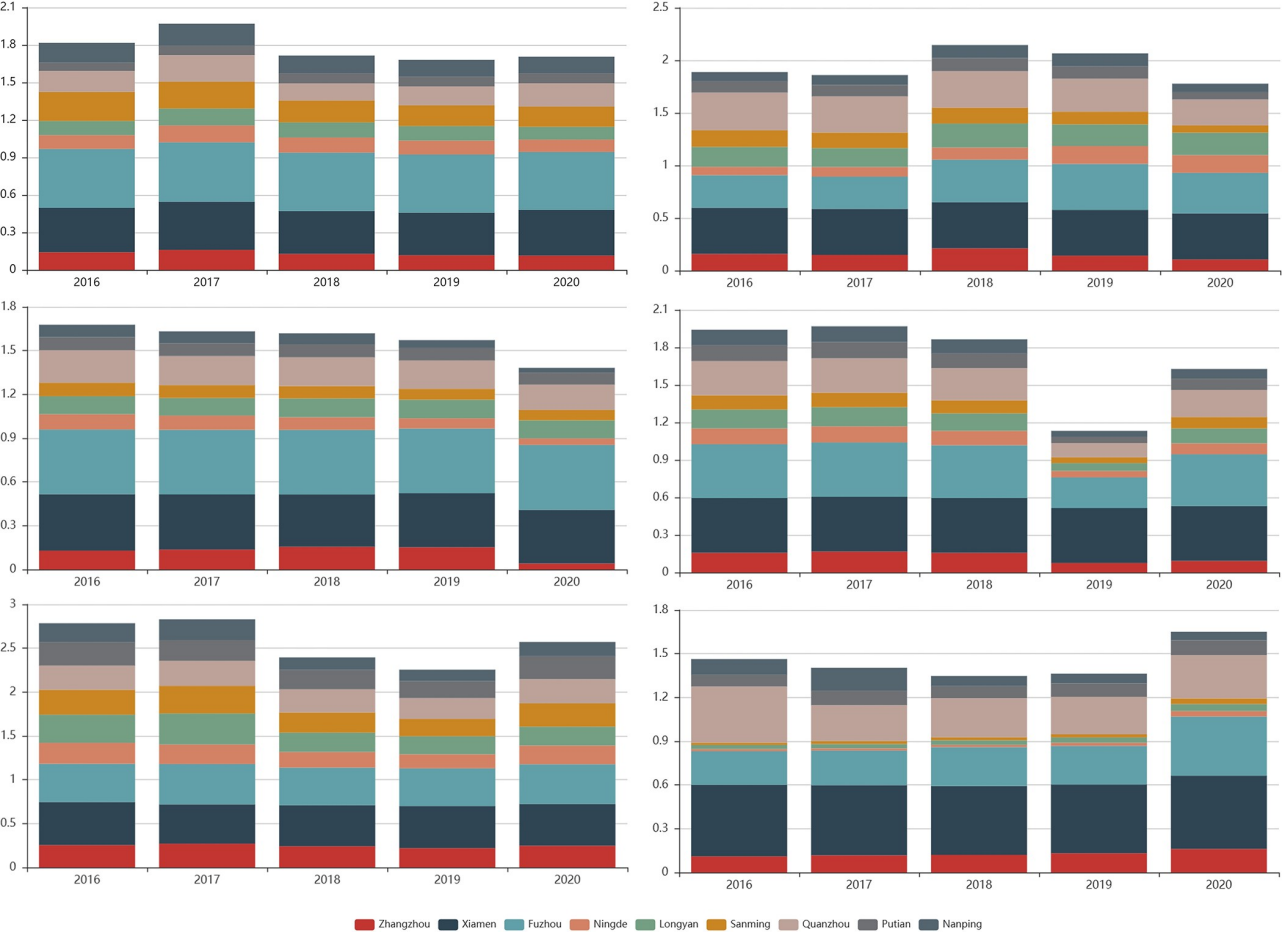
Close Degree of Progress (CDP) (spatial differences) in 2016–2020. (a) CDP of TR in 2016–2020. (b) CDP of ID1 in 2016–2020. (c) CDP of GD in 2016–2020. (d) CDP of ID2 in 2016–2020. (e) CDP of SD in 2016–2020. (f) CDP of OD in 2016–2020. (g) Type of cities.

Fujian Province’s topography features highlands in the northwest and lowlands in the southeast, creating a distinctive “mountain and sea” landscape. The evaluation of tourism resource development centers on two key aspects: natural resource conditions and the degree of tourism resource development. According to the calculation results (refer to Fig 4), Fuzhou and Xiamen exhibit robust tourism resource development capabilities, with an average Close Degree of Progress (CDP) level of 0.4435 and 0.3579, respectively. These cities showcase substantial progress in tourism resource development.

The Sanming region stands out for its ecotourism resources, with an average CDP level for tourism resources at 0.2250 between 2016 to 2017, reflecting its resource-rich status. However, from 2018 to 2019, this region’s ranking in terms of tourism resource development ability declined, potentially due to a less efficient utilization of resources.

Quanzhou experienced a significant boost in its resource development capacity from 2016 to 2017, with a remarkable 27% increase, attributed to the development of cultural tourism resources linked to the Silk Road culture during the Song and Yuan dynasties. Nanping, closely connected to Wuyi Mountain, a national park, possesses a strong foundational resource environment but lacks comprehensive resource development sharing, indicating untapped potential.

While other regions boast abundant mountain and sea resources, boast abundant, and their forms are relatively singular. This results in a low progress level in the allocation of tourism resources. The reasons for this include inadequate depth and strength in the protection and development of natural tourism resources, insufficient integration of cultural tourism resources from a regional perspective, a dearth of distinctive cultural tourism projects, and a lack of regional tourism brand influence.

Thus, it is essential to reinvigorate regional tourism resources based on conservation and inheritance, thus breathing new life into these resources to enhance the quality of tourism resources and boost the visibility of regional tourism.

#### Analysis of industry development (Fig 4B)

During the “13th Five-year Plan” period, Fujian Province adopted a rational tourism industry layout characterized by “three belts, three cores, and four zones”. The industry’s scale of gradually expanded, and the capacity for coordinated development among sectors improved. The added value of tourism and related industries in each region grew steadily, contributing more significantly to regional GDP.

As shown in Fig 4, Fuzhou and Xiamen shine as prominent leaders in the tourism industry, boasting average CDP values for industrial development of 0.4435 and 0.3723, respectively. These cities exhibit strong performance in areas such as government investment, integrated development of the tourism industry and related sectors, industrial innovation, the cultivation of new business models, and tourism cooperation with Taiwan. The tourism industry delivers substantial benefits in these areas.

In other cities, the CDP levels fall between 0.1933 and 0.2459, underscoring imbalances in the coordinated industrial development across the province. Cities like Zhangzhou and Nanping have faced challenges due to the impact of the pandemic, while their industrial development foundations need strengthening. Quanzhou and Longyan have established clear development positions, particularly in real estate industries, and are gradually improving policy support. Nevertheless, they grapple with issues like the absence of prominent industrial clusters with regional advantages, underdeveloped tourism transportation networks, inefficient inter-regional industrial linkage, and a lack of investment and financing mechanisms for the tourism industry, and unbalanced development of market factors.

Consequently, clear target positioning, an optimized industrial layout, resource industry integration, and the enhancement of policy facilities form the core concepts for tourism industry development.

#### Analysis of green development (Fig 4C)

Fujian Province boasts a remarkable 66.8% forest coverage rate, the highest in China, and a 95.8% proportion of I-III water quality in main river basins, rendering it a highly sought-after green destination. High-quality green development depends on ecological resources as its foundation, reinforced by effective system and skilled personnel. It is key that efforts are directed towards strengthening ecological environmental governance and expanding the tourism green industry chain. Compared to other dimensions, Fujian’s nine cities exhibit higher levels of green development as a whole.

Xiamen and Fuzhou lead the charge with impressive average CDP levels for green development, reaching 0.4725 and 0.4419, respectively. Xiamen, although small in urban space, has invested significantly in ecological protection and construction. Fuzhou has made substantial strides in green governance, with accomplishments like urban greenways and enhanced inland river system management, offering an improved quality of life for residents and tourists alike. Longyan, with the highest forest coverage rate in the province, is dedicated to forest health tourism, showcasing a robust regional green development level.

Other cities generally feature increasing financial investment in regional environmental protection, which bolsters the green governance capacity of regional cities. Discrepancies primarily stem from the need to enhance the regional ecological management mechanism, address imbalance in green development structures (including industrial structure, energy composition, transportation networks, land use patterns, etc.), and unlock the potential for translating tourism resource advantages into industrial strength.

#### Analysis of innovation development (Fig 4D)

Fujian Province has clearly defined its path for tourism innovation, intensifying investments in areas such as capital, talent, product and service innovation, marketing innovation, brand innovation, and the construction of intelligent cultural tourism. The “Digital Fujian” strategy guides the evolution toward digital cultural tourism, especially in Xiamen, which leads the change in high-quality tourism innovation development.

Xiamen’s high financial investment in scientific research, the creation of intelligent culture and tourism platform, and the influential branding of its coastal tourism all contribute to its leadership in innovation development, as indicated by the average CDP value of 0.4375 for regional innovation.

Fuzhou and Quanzhou are on par in terms of innovation development, boasting CDP values of 0.3688 and 0.3228, respectively. Fuzhou and Quanzhou focus on capital culture and Sea Silk culture, innovating cultural heritage tourism, enhancing tourism brand connotations, and optimizing tourism routes for high-quality, innovative regional tourism.

The other cities, including Longyan, Zhangzhou, Ningde, Putian, and Nanping, exhibit CDP levels of 0.2483, 0.2467, 0.2029, 0.2362, and 0.1778, respectively. These cities face distinct challenges in terms of innovation elements (such as policy, platforms, talent, capital, technology, etc.), a lack of tourism brand influence, slow development of official tourism platforms, regional disparities in the management level of smart scenic spots, and more.

Hence, technology empowerment serves as an essential tool for achieving high-quality, innovative tourism development.

#### Analysis of shared development (Fig 4E)

Fujian Province Adhere to the shared development concept of “Joint contribution by all and shared by all”, shared development of tourism in Fujian has made notable progress in areas like employment promotion, regional security, public facilities, urban space construction, industrial economic sharing in industrial sector, and the development of regional tourism.

Shared development, particularly in regional tourism, has served as a crucial driver of high-quality tourism development. Employment in tourism and related industries steadily increased over the years, particularly from 2018 to 2019, when the tourism industry expanded, leading to a significant rise in employment.

Most regions saw a consistent decline in the incidence of safety accidents year by year. Improvements were noted in healthcare and other public facilities. Nevertheless, the average CDP levels of shared development in Xiamen and Fuzhou stand at 0.4383 and 0.4291, respectively. These cities excelled in innovation in tourism cooperation mechanisms, comprehensive tourism planning, and extensive integration of the tourism industry.

Quanzhou achieved a CDP level of 0.2272, placing it in the middle range within Fujian Province. Quanzhou, driven by regional tourism development, focused on projects benefiting and enriching the local populace. In contrast, the remaining cities exhibited CDP levels below 0.1673, marked by challenges like limited awareness of regional shared development, a lack of top-level regional shared development mechanism, inadequate tourism and transportation systems, and the need to enhance spatial construction across the region to drive comprehensive industry integration.

#### Analysis of open development (Fig 4F)

Fujian Province, renowned as the homeland of overseas Chinese, has established stable tourist source market through its extensive overseas diaspora. In addition, a growing commitment to openness has attracted several high-profile international events to the province, such as the Fuzhou Sea International Film Festival, World Heritage Conference, and China Digital Summit. Xiamen, a pioneer in tourism openness, has hosted the Game of International Marathon, BRICS International Summit, the International Animation Festival, and more. These international events not only granted considerable foreign exchange income for the host regions but also realized resource spillover and benefits shared across the region.

Between 2016 and 2019, the number of inbound tourists and foreign exchange income in Fujian Province exhibited annual growth. Xiamen, as an early tourism hub and the host of several international activities and other open development resources, has achieved an average CDP level of open development at 0.4836, securing the top position in the province. The CDP of Quanzhou and Fuzhou stands at 0.2911 and 0.2814, respectively, positioning them at a medium level in Fujian Province, driven by the abundant resources of overseas Chinese towns and the substantial international influence of their cultural and tourism brands.

However, most other cities exhibit relatively lower levels of openness. For instance, Nanping, despite hosting the world-renowned “double heritage” resource, Wuyi Mountain, attracts a considerable inflow of tourism foreign exchange. Still, other counties and districts in Nanping show lower levels of openness, thereby affecting overall openness and development.

## Discussion and implications

Similar to the conclusions of studies on other regions [41,42], this study reveals a significant an imbalance and inadequacy in the high-quality development of tourism in Fujian Province. While certain cities along the southeastern coast of Fujian boast abundant tourism resources, high levels of openness to the outside world, a robust industrial foundation, and a strong capacity for innovation, the central-western and northern regions of the country excel in ecological resources and green development. However, they lag behind in terms of industry, innovation, and openness compared to the provincial average. Therefore, consistent with the research conclusion of Wang et al. [4], we believe that targeted and differentiated strategies are required to enhance the CDP level of high-quality tourism development and to balance the regional disparities. The specific measures include the following.

### Enhancing the quality of tourism resources throughout Fujian Province

During the “13th Five-year Plan” period, the tourism industry in Fujian embarked on an ecological-driven path of tourism development. In the “14th Five-year Plan”, Fujian’s tourism industry is poised to assume a pivotal role in the national strategy for achieving carbon neutrality and peak carbon emissions. To meet this goal, Fujian should focus on coordinating the development of green eco-tourism resources across the province, aiming to bolster resource reliance and provide fundamental support for high-quality tourism development. Here are the recommended measures:

1. Fujian should persist in strengthening ecological enhancements and refining ecological management mechanisms to address ecological vulnerabilities in areas like Ningde, Longyan, and Zhangzhou.
2. An essential focus should be on governance and risk mitigation. Leveraging digital technology, Fujian can improve its capacity to monitor the “carbon footprint” of tourism development and enable precise governance. This approach will curtail the release of “three wastes” associated with tourism, prevent forest fires, and minimize water (marine) environmental pollution. These efforts will collectively raise the overall quality of tourism resources in Fujian Province, emphasizing meticulous and comprehensive resource management in regions as Nanping, Sanming and Longyan.
3. Fujian should capitalize on natural and cultural tourism resources within cities, optimizing resource layout and integration while innovating tourism planning. Fujian should enrich the depth and breadth of tourism resources and explore regional differences, emphasizing creativity. This includes linking the high-quality cultural tourism resources that combine mountain and sea features in Fujian, as well as delving into the unique characteristics of She culture, Tea culture, Mazu culture, Sea Silk culture, and avoiding issues like homogeneity and seasonal dependence.

### Optimizing the supply of tourism industry in different regions

As the high-quality development of tourism advances, the tourism industry chain in Fujian Province is becoming increasingly diverse. However, it’s essential to ensure that the diverse supply aligns with regional tourism consumption demand, the regional industrial economy’s development, and the positioning of high-quality industrial development. To address this, the development approach for the tourism industry should be tailored to the unique characteristics and shortcomings of different regions within Fujian. This can be achieved through the following measures:

1. The government should focus on enhancing the top-level design of tourism industry development in Fujian Province. This entails establishing and refining various policies and mechanisms to support industrial growth and improve the array of services available for the tourism sector’s development.
2. Fujian Province should proactively nurture and drive the growth of several leading tourism enterprises. Leveraging preferential policies, these enterprises should be empowered to bolster their industrial financing capacity and promote differentiation within various regions and industries.
3. Fujian Province should expedite the adaptation and deep integration of the tourism industry with primary, secondary and tertiary sectors. This integration should be based on the distinctive advantages and positioning of regions that are already well-advanced in high-quality tourism development, such as Fuzhou, Xiamen, and Quanzhou, as well as the less-developed regions. It’s crucial to recognize that each region possesses distinct industrial strengths. When pursuing industrial integration in these diverse regions, it is vital to avoid spreading resources too thinly and instead prioritize focused refinement. Leading the way in this endeavor is the promotion of tourism integration with characteristic industries, while simultaneously pioneering the development of multi-level industrial integration spanning tourism, industry, trade, and culture in regions like Ningde and Zhangzhou. Additionally, there is a pressing need to advance the integration of tourism with health and agriculture in areas like Sanming, Putian as well as accelerate the integration of tourism with culture and forestry in regions such as Nanping and Longyan.

### Strengthening digital innovation in less-developed tourism areas

The process of digitization in tourism plays a crucial role in driving the speed of innovation and development in travel quality. However, several areas in Fujian Province are still sticking to scenic spots, lagging in terms of developing intelligent rural tourism area management, and intelligent services, particularly in the domains of intelligent marketing and intellectualization. In regions characterized by their mountainous terrain, such as Sanming, Longyan, Nanping, there are still evident challenges related to the slow adoption of intelligent management, services, and marketing in rural tourism areas. Therefore, expediting innovation in tourism and enhancing the quality of tourism experiences in these areas is of paramount importance.

To address these issues:

1. Fujian Province should substantially increase investment in innovation and development in less-developed high-quality tourism areas like Sanming, Longyan, and Nanping This investment should encompass a combination of supportive policies, diversified funding sources, investment in human resources, and technology imports.
2. Fujian Province should expand the coverage of the provincial tourism big data platform to encompass regions such as Sanming, Longyan, and Nanping, which are lagging in high-quality tourism development.
3. Fujian Province should enrich the content system of an intelligent tourism services in regions like Sanming, Longyan, and Nanping. This expansion should encompass various aspects, involving tourism supervision, publicity, and marketing, intelligent services for tourism areas (such as booking, tour guidance, information dissemination, and resource management), as well as measures to ensure tourism integrity and manage tourism-related risks effectively.

### Expanding open sharing in areas of high-quality tourism development

High-quality tourism development areas that embrace openness and sharing have a positive spillover effect that can stimulate the high-quality tourism development of other regions. To achieve this:

1. Regions with a high level of high-quality tourism development, such as Xiamen, Fuzhou, and Quanzhou, should emphasize the importance of shared development. They should establish various mechanisms and platforms for information sharing, cooperation, and management through government- business interaction. This will guide the orderly and shared development of the tourism market and facilitate policy sharing.
2. Enhance the high-standard and diversified tourist accommodation system in areas with high-quality tourism development, optimize the tourism transportation network across Fujian province, accelerate the comprehensive support for tourism public services in different regions, and improve the quality of comprehensive tourism space construction. This will enhance the experience of tourists in the province.
3. Fujian Province should introduce advanced tourism development concepts and models. Furthermore, the province should foster increased tourism economic exchanges and cooperation between regions like Xiamen, Fuzhou, and Quanzhou, enhancing the international influence of regional tourism brands that align with regional characteristics. Attention should be paid to the spillover effects of tourism development across regions, ultimately promoting open and shared high-quality tourism development in Fujian Province.

## Conclusions

Building upon existing relevant research, this paper defines the essence of high-quality tourism development by addressing the synergy of quantity and quality. It formulates an elemental model for high-quality tourism development and accordingly structures an evaluation index system tailored to Fujian Province. The computation of comprehensive weights for these indexes, as well as the Close Degree of Progress (CDP) for high-quality tourism development in Fujian Province, is achieved through the Entropy-weight method, Analytic Hierarchy Process (AHP), and Technique for Order of Preference by Similarity to Ideal Solution (TOPSIS) method. The following conclusions have been drawn:

1. A time series evolution analysis reveals a consistent and favorable overall development trend in the 9 cities of Fujian Province from 2016 to 2019. These years witnessed a steady upward trajectory marked by good development efficiency and results. In 2020, the impact of COVID-19 had a severe influence, resulting in varying degrees of development decline across all cities. This decline can be attributed to a sharp drop in the number of inbound tourists and tourist foreign exchange income. The most notable decline was observed in Zhangzhou city, which experienced a 44.7% reduction. In contrast, Fuzhou, Xiamen, Nanping, and Ningde saw smaller declines, ranging from 0.2% to 1.5%, indicating that these cities possess a robust foundation for high-quality tourism development. Putian and Longyan exhibited slight improvements in their CDP in 2020, primarily attributed to advancements in green development and industrial innovation.
2. The study period revealed an unbalanced regional development in the high-quality tourism development of Fujian Province. The gap between regions gradually narrowed from 2018 to 2019, with notable increases in the levels of green development, innovative development, and shared development across the board during these two years. Fuzhou and Xiamen have led high-quality tourism development, with Quanzhou ranking third. However, there exists a discernible gap in CDP levels between these leading cities and the others. Zhangzhou and Nanping experienced significant fluctuations in their comprehensive development ratings, indicating an overall development insufficiency.
3. From the perspective of dimensional analysis based on spatial differences, the nine cities in Fujian Province generally exhibit a higher level of green development. Concerning tourism resources, Fuzhou, Xiamen, Quanzhou, Sanming, and Nanping boast superior basic resource conditions, albeit with a shortfall in overall resource sharing. While other regions possess abundant mountain and sea resources, they lack diversification, feature single resource formats, and show low progress in resource allocation. Within the dimension of industrial development, Fuzhou and Xiamen demonstrate effectiveness in the tourism industry, whereas other cities have lower CDP levels for industrial development, suggesting a regional imbalance in the overall coordinated development of inter-regional industries within the province. As for innovation development, the average CDP levels in Longyan, Zhangzhou, Ningde, Putian and Nanping differs significantly from Fuzhou, Xiamen and Quanzhou, highlighting prominent deficiencies in innovation factors, such as policies, platforms, talents, funds, and technologies. In the dimension of shared and open development, Xiamen excels, while Fuzhou and Quanzhou perform at a medium level. Other cities exhibit lower CDP levels in shared and open development.

The innovation of this study can be summarized as follows:

1. This study introduces a novel perspective on tourism quality development by establishing a comprehensive framework for connotation analysis based on the new development concept. Different from previous research on the measurement of tourism development quality from the perspective of cultural and tourism integration [8], ecological civilization [41] and industrial integration [10], it constructs a tourism quality development model comprising six key elements: “support, means, orientation, motivation, purpose, and direction.” This model not only deepens our understanding of high-quality tourism development but also offers a valuable addition to existing research on tourism quality, particularly from the tourists’ perspective.
2. A large number of literatures have developed different evaluation index systems of high-quality tourism, but the results are difficult to compare [40]. The paper presents an indicator system and employs the CDP index, measured using Entropy-weight method, AHP, and TOPSIS method. These methodological tools are crucial for assessing the level of high-quality tourism development, analyzing spatial and temporal variations, and understanding the contributing factors.
3. This study quantitatively measures the CDP index for high-quality tourism development in Fujian Province, examining its temporal stage characteristics and spatial regional differences. These findings expand upon previous research perspectives on the spatial distribution pattern of high-quality tourism development [43,44].

Nonetheless, this paper has several limitations. Firstly, due to the data availability, he study utilizes a relatively limited dataset spanning only five years. Secondly, high-quality tourism development encompasses a wide range of factors, some of which are challenging to collect and quantify. Certain variables or indicators that could affect high-quality tourism development such as the Urban-rural tourism Engel coefficient ratio and the Usage of oil per unit output value in tourism, have been omitted due to data limitation. Thirdly, different from previous research [55,56], while this paper explores the elements driving high-quality tourism development, it does not delve deeply into the evolving relationship between quantitative and qualitative aspects and the underlying mechanisms.

In future studies, it is recommended to extend the data collection period and include additional indicators to enhance result reliability, provide a more comprehensive understanding of high-quality tourism development, and mitigate potential biases stemming from the exclusion of relevant data and indicators. Additionally, further theoretical research on evolutionary laws and mechanisms should be conducted to offer theoretical guidance for empirical research and practical applications.
